# Prediction of Host-Specific Genes by Pan-Genome Analyses of the Korean *Ralstonia solanacearum* Species Complex

**DOI:** 10.3389/fmicb.2019.00506

**Published:** 2019-03-15

**Authors:** Heejung Cho, Eun-Sung Song, Sunggi Heu, JeongHo Baek, Young Kee Lee, Seungdon Lee, Seon-Woo Lee, Dong Suk Park, Tae-Ho Lee, Jeong-Gu Kim, Ingyu Hwang

**Affiliations:** ^1^National Institute of Agricultural Sciences, Rural Development Administration, Jeonju, South Korea; ^2^National Institute of Crop Science, Rural Development Administration, Jeonju, South Korea; ^3^Department of Applied Biology, Dong-A University, Busan, South Korea; ^4^Department of Agricultural Biotechnology, Seoul National University, Seoul, South Korea

**Keywords:** *Ralstonia solanacearum* species complex, bacterial wilt, host specificity, genome, pan-genome, type III secretion system effectors

## Abstract

The soil-borne pathogenic *Ralstonia solanacearum* species complex (RSSC) is a group of plant pathogens that is economically destructive worldwide and has a broad host range, including various solanaceae plants, banana, ginger, sesame, and clove. Previously, Korean RSSC strains isolated from samples of potato bacterial wilt were grouped into four pathotypes based on virulence tests against potato, tomato, eggplant, and pepper. In this study, we sequenced the genomes of 25 Korean RSSC strains selected based on these pathotypes. The newly sequenced genomes were analyzed to determine the phylogenetic relationships between the strains with average nucleotide identity values, and structurally compared via multiple genome alignment using Mauve software. To identify candidate genes responsible for the host specificity of the pathotypes, functional genome comparisons were conducted by analyzing pan-genome orthologous group (POG) and type III secretion system effectors (T3es). POG analyses revealed that a total of 128 genes were shared only in tomato-non-pathogenic strains, 8 genes in tomato-pathogenic strains, 5 genes in eggplant-non-pathogenic strains, 7 genes in eggplant-pathogenic strains, 1 gene in pepper-non-pathogenic strains, and 34 genes in pepper-pathogenic strains. When we analyzed T3es, three host-specific effectors were predicted: RipS3 (SKWP3) and RipH3 (HLK3) were found only in tomato-pathogenic strains, and RipAC (PopC) were found only in eggplant-pathogenic strains. Overall, we identified host-specific genes and effectors that may be responsible for virulence functions in RSSC *in silico*. The expected characters of those genes suggest that the host range of RSSC is determined by the comprehensive actions of various virulence factors, including effectors, secretion systems, and metabolic enzymes.

## Introduction

The *Ralstonia solanacearum* species complex (RSSC) is a group of rod-shaped Gram-negative bacteria with polar flagella belonging to the Burkholderiaceae family of the Betaproteobacteria class. RSSC are soil-borne pathogens and can live for several years without a host. The bacteria invade host vascular tissues through injured roots or natural openings. Then, colonization and production of exopolysaccharide (EPS) in the stem block water transport in the xylem, resulting in wilting and death of the host plant (Denny, 2006).

*Ralstonia solanacearum* has an uncommonly broad host range, infecting more than 450 plant species that belong to more than 50 families, encompassing monocots and dicots and herbaceous and woody plants (Hayward, 1991; Wicker et al., 2007; Jiang et al., 2017). *R. solanacearum* are found in distinct geographical regions, which include tropical, subtropical, and warm and cool temperate areas across the six continents of Asia, Africa, Europe, and North and South America, and Oceania (Hayward, 1991; Denny, 2006). With their broad host range and wide geographical distribution, *R. solanacearum* have demonstrated great diversity in their genetic and phenotypic properties, and thus strains of this species have been designated as a species complex, the RSSC (Fegan and Prior, 2005; Genin and Denny, 2012). The RSSC group has been expanded to include *Ralstonia syzygii* and blood disease bacteria (BDB), which are closely related organisms (Taghavi et al., 1996).

The investigation of the genome of *R. solanacearum* began with the complete genome sequencing of strain GMI1000, which significantly advanced the study of pathogenicity by characterizing the molecular complexity of the organism (Salanoubat et al., 2002). Subsequently, the complete or draft genomes of many *R. solanacearum* strains with various host ranges became available. To date, there are 86 genomes of RSSC deposited in the National Center for Biotechnology Information (NCBI database in Aug 2018^1^). RSSC usually have a bipartite genome, with one chromosome and one megaplasmid; however, some strains (i.e., CMR15 and PSI07) carry an additional plasmid (Remenant et al., 2010). Whole-genome comparisons of sequenced genomes have confirmed the phylotype classification scheme of RSSC: phylotype I (GMI1000, FQY_4, EP1, and Y45), phylotype II (CFBP2957, IPO1609, K60, MolK2, Po82, and UW551), phylotype III (CMR15), and phylotype IV (PSI07, *R. syzygii* R24 and BDB R229). RSSC have been reclassified into three species, based on analyses of various biochemical properties and genomic comparisons, as follows: *R. solanacearum* (phylotype II strains), *Ralstonia pseudosolanacearum* (phylotype I and III strains), and *R. syzygii* (phylotype IV strains, including *R. syzygii* R24 and BDB R229) (Safni et al., 2014). RSSC is also divided into five biovars based on carbohydrate utilization, i.e. the ability to oxidize 3 disaccharides (lactose, maltose, and cellobiose) and 3 hexose alcohols (mannitol, dulcitol, and sorbitol) (Hayward, 1991).

A number of factors are responsible for the pathogenesis of RSSC: global regulatory transcription factors, EPS, plant hormones, host cell wall-degrading enzymes, adhesion/surface proteins, toxins, and oxidative stress resistance. Many of them are secreted in RSSC, and the bacterial pathogen mainly use type II (T2SS) and type III secretion systems (T3SS). Three types of T2SS, one orthodox system and two unorthodox systems, have been found in seven reference genomes, including those of phylotypes I, II, III, and IV (Li et al., 2016). T3SS is also crucial for bacterial virulence as the plant-inducible secretion machinery, which is encoded by the highly conserved *hrp* gene cluster in strains of phylotypes I, II, III, and IV (Li et al., 2016).

Type III secretion systems exports virulence factors directly into the host cells, and these injected proteins are called type III effector proteins (T3e) (Cunnac et al., 2004; Mukaihara et al., 2010). T3es of RSSC have been designated as Rip (*Ralstonia*-injected proteins), which include the Pop/AWR/Gala families (Peeters et al., 2013). These proteins have various functions in the invasion of host plants. For instance, the GALA family has F-box and leucine-rich repeat (LRR) domains that are required for full virulence (Angot et al., 2006; Kajava et al., 2008; Remigi et al., 2011). PopP family proteins work as avirulence proteins with acetyl transferase activity (PopP2) (Deslandes et al., 2003), and some AWR family effectors induce necrotic cell death in host plants (Sole et al., 2012). Likewise, T3e disrupts the homeostasis of host plants by disturbing signal transduction. Their defense system falters, leading to bacterial infection and death by wilting (Poueymiro and Genin, 2009). In a previous study, pan-genome analyses of 11 representative RSSC strains identified 94 Rips (Peeters et al., 2013). Individual RSSC strains possess around 60–75 effectors, and effector repertoire comparisons have revealed 32 core effectors in 10 strains. Most *rip* genes have a feature in their promoter region called a plant-inducible promoter (PIP) box, which is responsive to the T3SS transcriptional regulator HrpB (Cunnac et al., 2004).

A number of studies have investigated the host specificity of this highly vulnerable pathogen; however, because of the indefinite host range and bacterial nomenclature related to virulence, few systematic studies have clearly defined host specificity (Guidot et al., 2007; Cellier et al., 2012; Peeters et al., 2013; Ailloud et al., 2015). Nonetheless, the *rsa1* gene from strain SL2029 (phylotype IV biovar 2) has been described as a pepper-specific avirulence gene. The Rsa1 protein from strain SL2029 is specifically avirulent for pepper infection, and when this gene is introduced into the pepper pathogenic strain SL341 (phylotype I biovar 4), the SL341 with the *rsa1* gene becomes avirulent for pepper (Jeong et al., 2011).

In our previous study of the pathogenicity against potato, tomato, eggplant, and pepper, Korean RSSC were divided into four pathotypes. Since pathotypes reflect genetic traits of RSSC, we attempted to identify bacterial genes for host specificity of RSSC by adopting pan-genome analysis. In this study, we sequenced whole genome of 25 Korean RSSC strains and performed comparative genome analyses to present the candidate genes responsible for host specificity including T3es and pathogenesis related genes.

## Materials and Methods

### Strain Selection

We previously analyzed the genetic and pathogenic diversity of Korean RSSC using 93 strains isolated from samples of potato bacterial wilt throughout the country. To conduct in-depth analyses of the genetic relationships between RSSC and hosts, 25 isolates were selected based on their host range on solanaceous crop plants, with typical representative characters for each of the individual phylotypes (Cho et al., 2018).

### Genome Sequencing

For genomic DNA preparation, high-molecular-weight genomic DNA was prepared as previously described (Cho et al., 2018). Each bacterial genome was sequenced using the Pacific Biosciences’ Single Molecule Real Time (SMRT) Sequencing Technology with a 20 kb library, P6/C4 chemistry, and one SMRT cell running ( ^2^DNAlink, South Korea). *De novo* assembly was conducted using the hierarchical genome assembly process (HGAP ver. 2.3) workflow (Chin et al., 2013), including consensus polishing with Quiver, and the default parameters (Minimum Subread Length 500 bp, Minimum Polymerase Read Quality 0.8, and Minimum Polymerase Read Length 100 bp). After error correction based on the longest seed reads with shorter reads, sequences were assembled with error-corrected reads. Gene prediction was carried out using the Glimmer3 to predict coding sequences (CDSs), and RNAmmer-1.2 and tRNAscan-SE were used to identify rRNA and tRNA sequences in the assembled genome. The annotation of each CDS was performed using homology search against Blastall ver. 2.2.26.

### Genome Comparisons

For comparison with reference strains, the genome sequences of GMI1000, FQY_4, CMR15, PO82, and PSI07 were retrieved from the National Center for Biotechnology Information (NCBI) database and those of *R. syzygii* R24 and BDB R229 were obtained from the EMBL database (Table 1). To analyze the overall genome sequence similarity, the Orthologous Average Nucleotide Identity (OrthoANI) tool was used (Lee et al., 2016). Multiple genome alignments were performed using the Mauve software^3^.

**Table 1:** General genome features of the RSSC strains used in this study.

Strain^a^	Phyl-bv^b^	Contigs	Size (bp)	GC (%)	CDSs	rRNA	tRNA	Genome accession^c^
SL2312	IV-2	2	5,521,456	66.4	5,079	9	56	CP022796, CP022797
SL2064	IV-2	2	5,473,607	66.4	5,169	9	55	CP022798, CP022799
SL3022	IV-2	2	5,605,251	66.3	5,376	9	55	CP023016, CP023017
SL3175	IV-2	2	5,555,993	66.4	5,170	9	54	CP022788, CP022789
T11	IV-2	2	5,450,627	66.4	5,143	9	56	CP022776, CP022777
T12	IV-2	2	5,520,985	66.4	5,189	9	56	CP022774, CP022775
T51	IV-2	2	5,400,849	66.4	5,074	9	55	CP022770, CP022771
T82	IV-2	2	5,521,457	66.4	5,071	9	56	CP022763, CP022764
T95	IV-2	2	5,474,514	66.4	5,146	9	55	CP022761, CP022762
T98	IV-2	2	5,555,978	66.4	5,172	9	54	CP022759, CP022760
T101	IV-2	2	5,521,368	66.4	5,086	9	56	CP022757, CP022758
SL2330	I-3	2	5,674,600	67	5,242	12	58	CP022794, CP022795
SL3755	I-3	2	5,792,854	66.9	5,413	12	58	CP022782, CP022783
T25	I-3	2	5,715,510	67	5,805	12	58	CP023014, CP023015
T110	I-3	2	5,642,243	67.1	6,811	12	57	CP023012, CP023013
SL2729	I-4	2	5,703,338	67	5,304	12	58	CP022792, CP022793
SL3103	I-4	2	5,618,133	67	5,257	12	58	CP022790, CP022791
SL3300	I-4	2	5,903,911	66.8	5,482	12	58	CP022786, CP022787
SL3730	I-4	2	5,686,064	67	5,348	12	58	CP022784, CP022785
SL3822	I-4	2	5,971,831	66.8	5,558	12	59	CP022780, CP022781
SL3882	I-4	2	6,025,869	66.8	5,594	12	59	CP022778, CP022779
T42	I-4	2	5,497,698	67	5,133	12	57	CP022772, CP022773
T60	I-4	2	6,015,554	66.8	5,588	12	59	CP022768, CP022769
T78	I-4	3	6,147,432	66.7	5,807	12	59	CP022765, CP022766,CP022767
T117	I-4	2	5,807,463	66.9	5,378	12	59	CP022755,CP022756
PSI07	IV-2	3	5,605,618	66.32	4,810	9	54	FP885906.2, FP885891.2
*R*. *syzygii* R24	IV	7	5,423,991	65.87	4,865	6	50	EMBL FR854086 – FR854092
BDB R229	IV	27	5,158,998	66.44	4,614	8	67	EMBL FR854059 – FR854085
CMR15	III	3	5,590,372	66.79	4,890	12	59	FP885895.1, FP885896.1, FP885893.1
CFBP2957	IIA-2	1	3,417,386	66.44	3,158	9	53	FP885897.1
Po82	IIB-2	2	5,430,263	66.67	4,745	9	54	CP002819.1, CP002820.1
IPO1609	IIB-2	10	5,318,522	64.85	4,659	6	31	NZ_CDGL000000000.1
GMI1000	I-3	2	5,810,922	66.98	5,055	12	57	AL646052.1, AL646053.1
FQY_4	I-4	2	5,805,250	66.81	5,068	12	51	CP004012.1, CP004013.1


*^a^Strain T78 had third circular plasmid of 128,742 bp, 156 CDSs, and no rRNA and tRNA. ^*b*^Phylotype-biovar. ^*c*^NCBI genome accession numbers, except *R. syzygii* R24 and Blood Disease Bacterium (BDB) R229 genome, which were EMBL genome accession numbers.*

### Analysis of Clusters of Orthologous Groups and Pan-Genome Orthologous Groups

For comparative genome analyses, clusters of orthologous group (COG) and pan-genome orthologous group (POG) analyses were performed using the Chunlab pipeline (^4^South Korea) (Chun et al., 2009). Gene prediction and annotation were analyzed using the Server for Rapid Annotations using Subsystem Technology (RAST) (Aziz et al., 2008). The annotation of each CDS was conducted by homology search against the Swiss-Prot, EggNOG 4.1, SEED (the database and infrastructure for comparative genomics), and Kyoto Encyclopedia of Genes and Genomes (KEGG) databases.

### Effectors Prediction

The effectors of T3es of the Korean RSSC strains were identified using the RalstoT3E server^5^ (Peeters et al., 2013).

### Genome Submission

The genome sequences of 25 Korean RSSC strains were deposited in the NCBI database and the accession numbers are listed in Table 1.

## Results

### General Genomic Features of the RSSC Strains

High-quality genomes of the Korean RSSC strains were constructed using PacBio long read sequencing data. The general genomic features of the RSSC strains used in this study are summarized in Table 1. The newly sequenced 25 Korean RSSC genomes were complete and contained two contigs: one for the chromosome and another for a megaplasmid, except for T78 strain, which contained one chromosome, one megaplasmid, and one small plasmid.

All 25 strains analyzed in this study belong to phylotype I and IV, the assembled genome sizes were 5.4–6.15 Mbp and the GC contents were 66.4–66.9%. The number of predicted genes was 5,071–6,811, that of rRNA genes was 9 or 12, and that of tRNA genes was 54–59. The genome comparison revealed several differences between the genomes of phylotype I and phylotype IV, as follows: the average genome sizes of phylotype I and IV were 5.8 and 5.5 Mbp, the average GC contents were 66.91 and 66.39%, the average number of predicted CDSs was 5,551 and 5,152, the number of rRNA genes was 12 and 9, and the average number of tRNA genes was 58 and 55, respectively. All genomic features (genome size, GC content, and number of CDSs, rRNAs, and tRNAs) of the phylotype I strains were larger than those of the phylotype IV strains, except for the T42 strain, which was similar in genome size and CDS number to the phylotype IV strains.

### Comparative Genomics: OrthoANI Analyses and Multiple Genome Alignments

**FIGURE 1:**
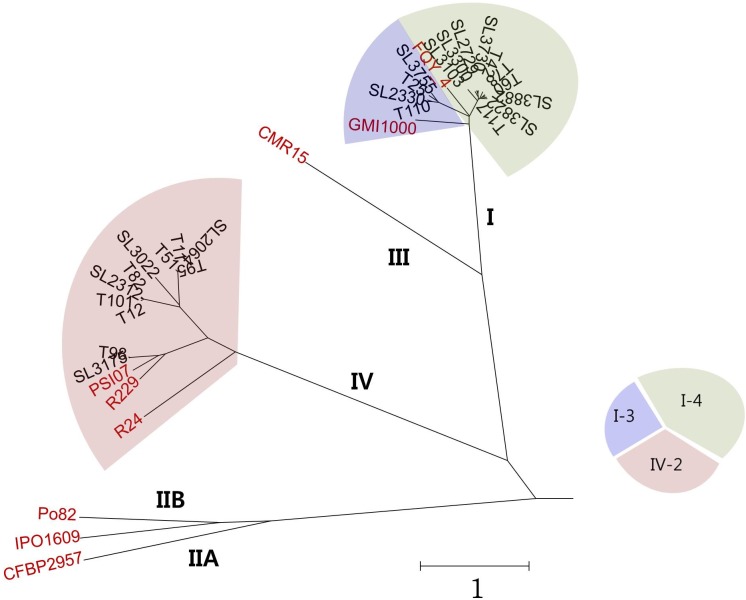
Phylogenetic tree prepared using genomes of 25 Korean and 9 reference RSSC strains based on OrthoANI values.

To compare the overall similarity of the 25 Korean genomes and 9 reference genomes, the OrthoANI values were calculated (Supplementary Table S1) and are presented with the phylogenetic tree in Figure 1. In this tree, all Korean phylotype I strains clustered with the phylotype I reference strains GMI1000 (biovar 3, isolated from tomato in French Guyana) and FQY_4 (biovar 4, isolated from bacterial wilt in a nursery in China) (Salanoubat et al., 2002; Cao et al., 2013). Among the Korean phylotype I strains, four biovar 3 isolates (SL2330, SL3755, T25, and T110) clustered more closely with GMI1000, and ten other biovar 4 isolates (SL2729, SL3103, SL3300, SL3730, SL3822, SL3882, T42, T60, T78, and T117) clustered more closely with FQY_4. Eleven Korean phylotype IV strains (SL2064, SL2312, SL3022, SL3175, T11, T12, T51, T82, T95, T98, and T101) clustered with the phylotype IV strains of PSI07 (from tomato in Indonesia), BDB R229 (from banana in Indonesia), and *R. syzygii* R24 (from clove in Indonesia), which originated from the Indonesian region. Among the Korean phylotype IV strains, SL3175 and T98 were closer to reference strains PSI07 and BDB R229.

**FIGURE 2:**
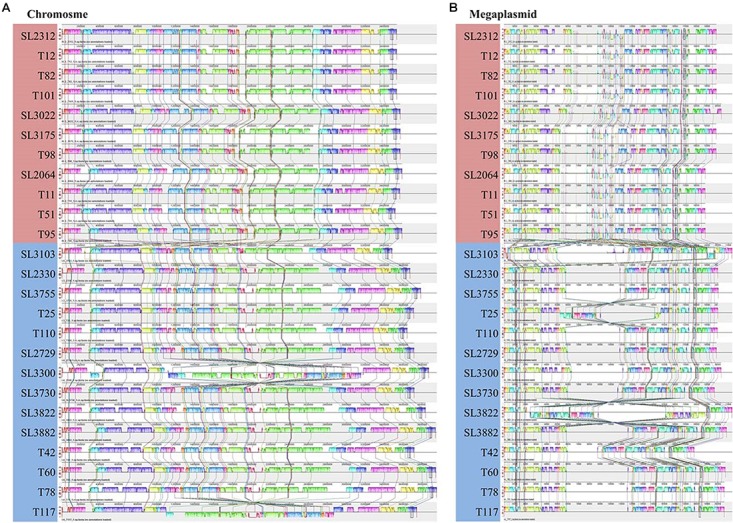
Multiple genome alignment for 25 Korean RSSC strains produce using Mauve software. The sequences of chromosome **(A)** and megaplasmid **(B)** were aligned. The red and blue boxes represent phylotype IV and I, respectively. Colored lines between genomes represent rearrangements or inversions.

To demonstrate the consistency or variation among the Korean RSSC genomes, multiple genome alignment was performed using the Mauve tool (Figure 2). This alignment revealed that eleven phylotype IV-biovar 2 (hereafter, IV-2) strains were co-linear along the chromosomes and the megaplasmids. For phylotype I strains, the phylotype I-biovar 3 (I-3) and phylotype I-biovar 4 (I-4) strains were generally similar to each other with respect to their genome organization; however, some strains had a genetic inversion, i.e., SL3300 and T117 had an inversion in the middle of the chromosome, and T25 and SL3822 had a large inversion in the megaplasmid. Between the phylotype I and IV strains, the gene organization revealed many rearrangements, particularly in the megaplasmid.

### COG Distribution Between RSSC Strains

**FIGURE 3:**
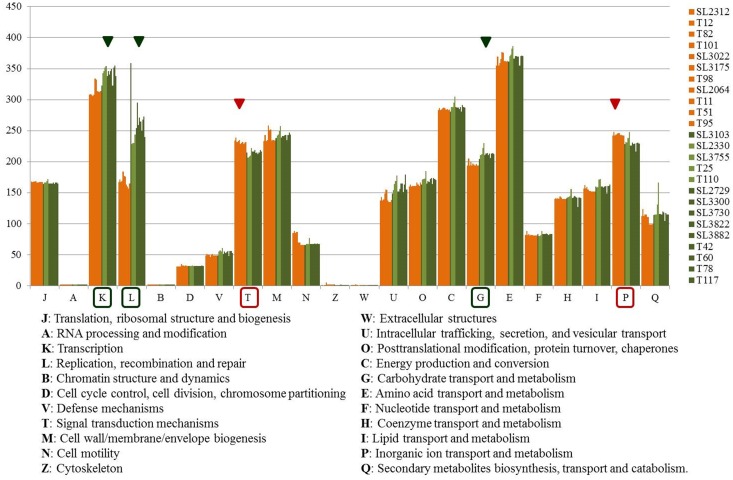
Graph of COG functional categories of 25 Korean strains. Orange color represents phylotype IV-biovar 2 strains, and olive and green colors represent the phylotype I-biovar 3 and phylotype I-biovar 4 strains. The red triangle and box indicate the category with more genes in phylotype IV than in phylotype I, and the green triangle and box indicate the category with more genes in phylotype I than phyloytpe IV. Category S, genes of unknown function, is excluded from the graph.

To compare the distribution of functional genes between the RSSC strains in relation to bacterial virulence and potential host specificity, the functional categories of COG were analyzed (Figure 3 and Supplementary Table S2). Among the predicted CDSs, about 70% of the genes were classified into one of the 22 COG categories and about 30% were of unknown function. Aside from the genes of unknown function (S), the largest functional group was the amino acid transport and metabolism group (E), which contained an average of 367 genes, followed by the transcription group (K), which contained an average of 330 genes, and the energy production and conversion group (C), which contained an average of 286 genes. COGs were distributed differently between the phylotype I and IV strains. The phylotype I strains had more genes than the phylotype IV strains in the categories of transcription (K); replication; recombination and repair (L); intracellular trafficking, secretion, and vesicular transport (U); carbohydrate transport and metabolism (G); and secondary metabolite biosynthesis, transport, and catabolism (Q). The phylotype IV strains had more genes than the phylotype I strains in the categories of signal transduction mechanisms (T) and inorganic ion transport and metabolism (P).

### Candidate Host-Specific Genes for Bacterial Virulence

To identify genes responsible for host specificity, comparative genome analyses were performed by comparing functional genes among the four pathotypes. Previously, Korean RSSC strains isolated from samples of potato bacterial wilt were divided into four different pathotypes based on tests against four *solanaceae* plants: potato, tomato, eggplant, and pepper. The classifications were only pathogenic on potato (P); pathogenic on potato and tomato (PT); pathogenic on potato, tomato, and eggplant (PTE); and pathogenic on potato, tomato, eggplant, and pepper (PTEPe) (Cho et al., 2018).

Using pan-genome analyses of the Korean isolates, we identified a number of candidate genes expected to contribute to host specificity (Table 2) and the POGs are listed in Supplementary Tables S3–S5. A total of 128 genes were only found in four tomato-non-pathogenic strains. Most of these genes encoded proteins with hypothetical functions and a few of the functionally designated genes were related to mobile elements, such as bacteriophage infection or insertional elements (Supplementary Table S3). Three genes revealed homology with a gene encoding the clustered regularly interspaced short palindromic repeat (CRISPR) proteins, Cas9, Cas1, and Cas2 (Supplementary Figure S1A). CRISPR is an adaptive immune system in prokaryotes that provides protection against mobile genetic elements (viruses, transposable elements, and conjugative plasmids). Four genes showed homology with a gene encoding Mu-like prophage proteins (Supplementary Figure S1B), and one capsid protein and another Mu-like virus tape-measure protein were identified by the analyses. In addition, two copies of insertional element IS476 were also identified in the analyses (Supplementary Figure S1C). In the tomato-pathogenic group, these bacteria shared 8 genes. Among them, three genes encoded components of the type II secretion system (T2SS) and next one gene encoded RhsB (rearrangement hotspot), which is a probable deoxyribonuclease (Table 2, Supplementary Figure S2, and Supplementary Table S3).

**Table 2:** Specific gene numbers of RSSC strains that are pathogenic and nonpathogenic toward tomato, eggplant, and pepper.

Strains	Phylotype-biovar	Original Host	Pathotype^a^	Specific genes		
				Tomato	Eggplant	Pepper
SL2312	IV-2	Potato	P			
T12	IV-2	Potato	P			
T82	IV-2	Potato	P	128	5	1
T101	IV-2	Potato	P			
SL3022	IV-2	Potato	PT			
SL3175	IV-2	Potato	PT			
T98	IV-2	Potato	PT			
SL2064	IV-2	Potato	PTE			
T11	IV-2	Potato	PTE			
T51	IV-2	Potato	PTE			
T95	IV-2	Potato	PTE			
SL3103	I-4	Potato	PTE			
SL2330	I-3	Potato	PTEPe			
SL3755	I-3	Potato	PTEPe			
T25	I-3	Potato	PTEPe	8	7	34
T110	I-3	Potato	PTEPe			
SL2729	I-4	Potato	PTEPe			
SL3300	I-4	Potato	PTEPe			
SL3730	I-4	Potato	PTEPe			
SL3822	I-4	Potato	PTEPe			
SL3882	I-4	Potato	PTEPe			
T42	I-4	Potato	PTEPe			
T60	I-4	Potato	PTEPe			
T78	I-4	Potato	PTEPe			
T117	I-4	Potato	PTEPe			


*^a^Pathotype: (P) only pathogenic on potato, (PT) pathogenic on potato and tomato, (PTE) pathogenic on potato, tomato, and eggplant, and (PTEPe) pathogenic on all tested crops – potato, tomato, eggplant, and pepper.*

Regarding eggplant, seven eggplant-non-pathogenic strains shared 5 genes (Supplementary Table S4). Four of them encoded hypothetical proteins and one was similar to a gene encoding a putative RipA, which is a transcriptional regulator for type III secretion with a helix-turn-helix DNA binding motif (Supplementary Figure S3). For eggplant infection, it was predicted that 7 genes were shared. None of these genes were similar to genes of known function.

For pepper, the phylotype I strain SL3103 belonged to the pepper-non-pathogenic group, unlike other phylotype I strains, all of which were pathogenic on pepper plants. As a result, we were able to determine the candidate genes related to organic metabolisms rather than effectors. Twelve pepper-non-pathogenic strains (eleven phylotype IV and one phylotype I strains) shared 1 gene, whose function was unknown, and thirteen pepper-pathogenic strains (all phylotype I strains) shared 34 genes, including genes involved in aromatic compound metabolism (Supplementary Figure S4 and Supplementary Table S5). Among them, 12 genes (from POG_2330_01480 to POG_2330_01491) produced a cluster related to aromatic compound metabolism (Supplementary Table S5). This cluster was highly homologous with the *dhb* gene cluster from *Pseudomonas reinekei* MT1: *dhbA* (67% identity with POG_2330_01480), *dhbB* (64% identity with POG_2330_01481), *dhbC* (65% identity with POG_2330_01482), *dhbD* (47% identity with POG_2330_01483), *dhbE* (42% identity with POG_2330_01484), *dhbF* (60% identity with POG_2330_01485), *dhbG* (66% identity with POG_2330_01486), *dhbH* (83% identity with POG_2330_01487). This *dhb* cluster encodes genes that participate in 2,3-dihyroxybenzoate (2,3-DHB) aromatic ring degradation via the *meta*-cleavage pathway (Marin et al., 2012).

### T3SS Effectors

**FIGURE 4:**
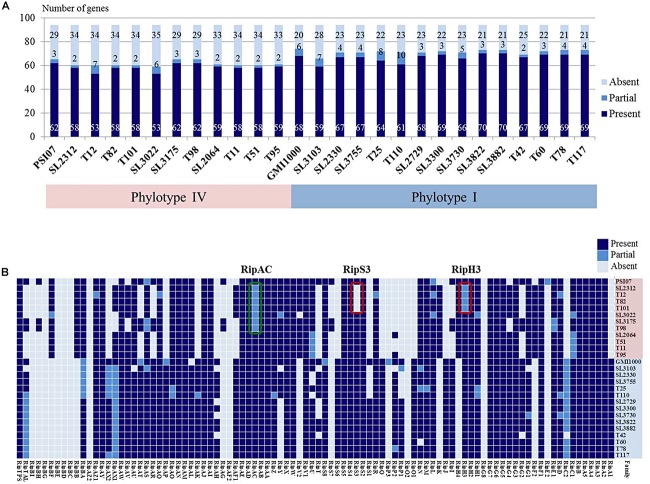
**(A)** Graph of predicted effector gene numbers. The colors represent as follows: dark blue, presence of a gene; blue, partial gene; light blue, absence of a gene. **(B)** Distribution of the T3e genes in 25 Korean strains and the reference GMI1000 and PSI07 strains. Red boxes represent tomato-non-pathogenic strains and green box represent eggplant-non-pathogenic strains.

Type III secretion systems is deeply involved in pathogenicity and RSSC carries abundant T3es that are secreted through the T3SS (Peeters et al., 2013). Therefore, we predicted the T3es of the Korean RSSC using the RalstoT3E annotation server and analyzed effectors related to host range on four Solanaceae plants (Figure 4). Of a total of 94 T3e repertoires of RSSC, 82 effectors were identified as full or partial forms and 12 effectors were absent (Figure 4A). While a total of 30 effectors were present in all sequenced Korean strains, 8 were present only in phylotype I and 6 were found only in phylotype IV (Figure 4B). Regarding the sharing of effectors, it was shared 68 effectors in four I-3 strains, 70 in ten I-4 strains, and 70 in eleven IV-2 strains. In general, phylotype I strains had more effectors than phylotype IV strains and the distributions of each effector revealed a more conserved patterns in phylotype I strains than in phylotype IV strains. In the case of SL3175 and T98 of phylotype IV, revealed different distribution pattern compared to the other phylotype IV strains, which this clustering was consistent with the genomic phylogenetic tree (Figure 1).

**FIGURE 5:**
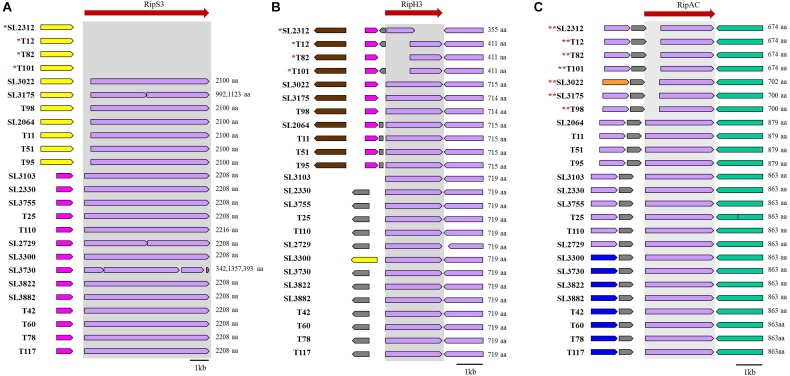
Genetic regions of RipS3 **(A)**, RipH3 **(B)**, and RipAC **(C)**. **^∗^**non-pathogenic strains for tomato. **^∗∗^**non-pathogenic strains for eggplant.

Next, we investigated the presence of effectors related to host specificity for tomato, eggplant, or pepper plant infections. Among them, we identified three host-specific effectors: two for tomato (RipH3 and RipS3) and one for eggplant (RipAC). The genetic loci of the genes encoding these proteins are depicted in Figure 5. The gene *ripS3* (also known as SKWP3) was present in all 21 tomato-pathogenic strains, but was absent in the four tomato-non-pathogenic strains (SL2312, T12, T82, and T101). The function of RipS3 is unknown; however, it contains the nucleotidyl transferase (NT) domain of the RelA- and Spo-like protein (Supplementary Figure S5A). The gene *ripH3* (HLK3) was present as a partial form with a truncated N-terminal or C-terminal region in the tomato-non-pathogenic strains, whereas it was present in a complete form in tomato-pathogenic strains. The *ripH3* gene contained a PIP box in the promoter region, but did not exhibit any known functional domain. In the case of RipAC (PopC) in strains pathogenic for eggplant, the gene was different from that of non-pathogenic strains, which had another type of *ripAC* (Supplementary Figure S5B). In the *ripAC* gene, it was difficult to find DNA sequence similarity between eggplant-pathogenic and non-pathogenic strains; however, there was 34% homology in the amino acid sequences, which had 17 LRR motifs and 10 LRRs in common with each other.

## Discussion

*Ralstonia solanacearum* species complex is a sophisticated complex of plant pathogens with an unusually broad host range and wide geographical distribution. We identified genes that are relevant to host specificity by analyzing whole genomes of 25 Korean RSSC strains isolated from potato bacterial wilt and performing *in silico* genome-wide comparison of four different pathotypes of RSSC strains.

The isolated Korean potato bacterial wilt strains belonged to phylotypes I or IV, and phylotype I strains showed destructive pathogenicity, not only on potato, but also on tomato, eggplant, and pepper plants (Cho et al., 2018). Previously, it had been suggested that phylotype I strains may have evolved from phylotype IV strains (Wicker et al., 2012; Li et al., 2016). The genomes of phylotype I strains possess distinctive features compared to those of the other representative phylotype II (Po82, IPO1609), III (CMR15), and IV strains (PSI07, R24, and R229): large genome size, high GC content, and larger numbers of rRNA and tRNA genes. From these genomic features, we suppose the possibility that phylotype I strains have accepted foreign DNA fragments to adapt to various environments resulting in increased GC content and genome size, and among the imported fragments, there might be various virulence factors that enable these strains to infect new hosts.

**FIGURE 6:**
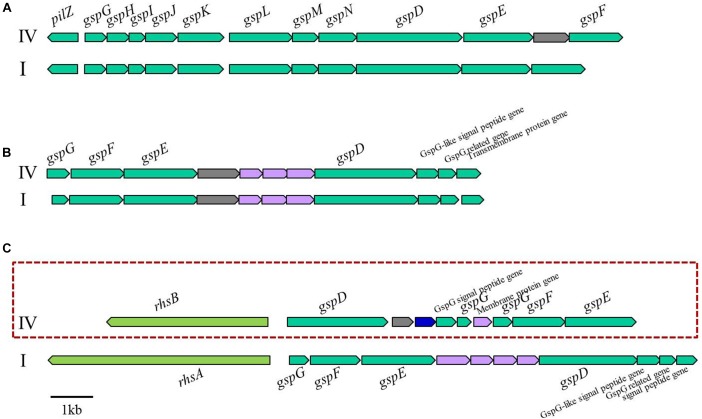
Genetic organization of three T2SS gene clusters in phylotype IV and I strains of RSSC **(A)** orthodox T2SS cluster, **(B)** and **(C)** unorthodox T2SS cluster. IV, phylotype IV; I, phylotype I. The T2SS gene cluster in the red box did not exist in the strains of tomato non-pathogens (SL2312, T12, T82, and T101).

When we compared the genomes to identify genes responsible for host specificity, we found specific genes from the tomato pathogenic strains: two T3es (*ripS3* and *ripH3*), a set of T2SS, and an adjacent *rhsB* (Figure 6). The function of RipS3 has not been reported; however, RipS3 has the nucleotidyl transferase domain of RelA- and SpoT-like ppGpp synthetases and hydrolases, and belongs to the PRK09169 superfamily (Supplementary Figure S5A). The ppGpp works as an alarmone, which integrates general stress responses, such as starvation, heat shock, and oxidative stress in bacteria and plants (Van der Biezen et al., 2000; Braeken et al., 2006; Wang et al., 2016). We speculate that effector RipS3 is translocated into host plant cells and may disturb the host stress response mediated by the ppGpp. The other effector, RipH3, possesses a PIP box but no known functional motif. The *ripH3* gene was partially present in the tomato-non-pathogenic strains (Figure 5), and this feature is consistent with a previous report that a triple deletion mutant of *ripH1*-*H3* (HLK1–3) was significantly impaired with respect to tomato infection (Chen et al., 2014).

Type II secretion systems (T2SSs) have been identified in bacteria belonging to the alpha, beta, gamma, and delta-proteobacteria, and are encoded by 12 to 16 genes, of which the core components are named as *gsp* (general secretory proteins) (Abby et al., 2016). Substrates of T2SS are recruited and transported as fully folded and often oligomeric proteins (Gu et al., 2017). It has been reported that RSSC possessed three types of T2SS: one is the orthodox system constructed out of 12 components, and the others are the unorthodox systems, possessing 7 core genes (Li et al., 2016). In our study, the tomato-non-pathogenic strains were lacking one set of unorthodox T2SS and the adjacent *rhsB* gene (Figure 6). Because T2SS mutants were impaired in colonization and proliferation *in planta* (Kang et al., 1994), this feature appears to relate one set of the T2SS deficiency with the tomato-non-pathogenic trait. In addition, the *rhsB* gene, which was located next to the T2SS-encoding gene cluster, was also absent in the tomato-non-pathogenic strains. Rhs protein possesses YD-peptide repeats, which play a role in the bacterial-eukaryotic host cell interaction, and it also carries nuclease domain to degrade target cell DNA (Koskiniemi et al., 2013). In *Dickeya dadantii* 3937, Rhs protein worked as a DNase toxin to inhibit neighboring cell growth in a contact-dependent manner, and its translocation to host cells provided evidence that Rhs may be exported through a type VI secretion system (T6SS) (Koskiniemi et al., 2013). Although it is a distinctive genetic feature of tomato-pathogenic strains, further experiments are required to define the mechanisms of T2SS and RhsB in tomato infection.

Our pan-genome analyses to identify host-specific genes for bacterial virulence revealed that a number of genes encoding hypothetical protein with unknown function and mobile element or phage-related genes are present in four pathotypes of Korean RSSC (Supplementary Tables S3–S5). Functional studies of each gene identified by *in silico* analysis should be performed to define their involvement in bacterial virulence in a host-specific infection and virulence. It would be also interesting to investigate if mobile element or prophages can contribute to host specific virulence of each pathotypes by disrupting certain avirulence- or virulence-related function of RSSC strains.

In analyses using the RalstoT3E prediction system, we identified the RipAC for eggplant-specific effector. RipAC was previously called as PopC and has LRR motifs, which are expected to interact with some host proteins (Peeters et al., 2013). The *popC* (*ripAC*) gene constitutes an operon with *popA* (*ripX*) and *popB* (*ripAB*) expressed by a promoter containing a PIP box (Guéneron et al., 2000). This *popABC* operon was located next to the *hrp* gene cluster, and the encoded PopA (harpin), PopB (NLS motif), and PopC (LRR motifs) proteins were secreted by T3SS. In the RalstoT3E database, the names of these effectors were assigned as RipX (PopA), RipAB (PopB), and RipAC (PopC) (Peeters et al., 2013). Interestingly, the RNA expressions of these genes were upregulated in tomato plants (Ailloud et al., 2016), but Tn5-B20-inserted mutants of these genes were still pathogenic on tomato (Marenda et al., 1998). In general, the Korean RSSC strains carried the similar gene organization with previously reported RSSC strains for *popABC* operon driven by a promoter with a PIP box and adjacent *hrp* cluster. However, while *popA* (*ripX*) and *popB* (*ripAB*) were present in all strains, *popC* (*ripAC*) was not; the *popC* gene of most strains encoded a protein with 17 LRR motifs, like that of the GMI1000 strain, but 7 eggplant-non-pathogenic strains carried a smaller *popC* gene encoding 10 LRR motifs, like that of R24 and BDB 225 (Supplementary Figure S5B). These results suggest that *popC* (*ripAC*) may be involved in eggplant-infection of RSSC.

The *dhb* gene cluster homologs were identified as pepper-specific candidate virulence genes. The metabolite 2,3-dihydroxybenzoate (DHB) works as an isochorismate-derived secondary metabolite in plants (Bartsch et al., 2010), or a key intermediate of several siderophores: enterobactin (*Salmonella enterica* and *Escherichia coli*), anguibactin, and vanchrobacin (*Vibrio anguillarum*) (Raymond et al., 2003; Li and Ma, 2017). Siderophores are low molecular weight iron-chelating molecules that facilitates iron uptake in many gram-negative bacteria. Iron uptake systems are critical for the function of some pathogens to infect host plants to lead to disease (Wolf and Crosa, 1986). In the pepper-non-pathogenic RSSC strains, the *dhb* gene cluster homologs and other related genes were absent. This deficiency appeared to affect the ability of the bacteria to infect pepper.

A previous study identified a pepper-specific avirulence gene *rsa1*, which confers avirulence to a pepper-pathogenic strain, from a potato pathogenic SL2029 (Jeong et al., 2011). In our study, this *rsa1* gene was present in the genome of all pepper non-pathogenic phylotype IV strains of Korean RSSC as expected. However, the *rsa1* gene was not identified as a host-specific avirulence gene in comparative analysis. This was because one of phylotype I strains, SL3103, does not carry *rsa1* gene in its genome while this strain was pepper non-pathogenic unlike to other phylotype I strains. It is likely that the strain SL3103 is pepper non-pathogenic due to the absence of *dhb* gene cluster (Supplementary Figure S4 and Supplementary Table S5), while other phylotype I strains carry *dhb* gene cluster.

Our extensive comparative genomic analyses uncovered several genes associated with the pathogenicity of RSSC on different crops. It is likely that the host-specificity of RSSC will be a function of the comprehensive actions of various virulence factors, effectors, secretion systems, and metabolic enzymes. Although further biological functions of these genes should be determined, these data contribute to expand our understanding on the host specificity of RSSC.

## Data Availability

The whole genome sequences of 25 Korean RSSC strains can be found in the NCBI GenBank and the accession numbers are listed in Table 1.

## Author Contributions

HC, E-SS, DP, J-GK, and IH conceived and designed the study. YL, SL, and S-WL provided the RSSC isolates and related information. HC and E-SS carried out the experiments. JB and T-HL assembled and analyzed the genomes. HC and IH analyzed and interpreted the data. HC, SH, and S-WL prepared the manuscript.

## Conflict of Interest Statement

The authors declare that the research was conducted in the absence of any commercial or financial relationships that could be construed as a potential conflict of interest.

## Footnotes

1 https://www.ncbi.nlm.nih.gov

2 www.dnalink.com

3 http://darlinglab.org/mauve/mauve.html

4 http://www.chunlab.com

5 https://iant.toulouse.inra.fr/bacteria/annotation/site/prj/T3Ev3
