# Supplementary material for: Prediction of Host-Specific Genes by Pan-Genome Analyses of the Korean Ralstonia solanacearum Species Complex
Source: Front Microbiol. 2019 Mar 15;10:506. doi: 10.3389/fmicb.2019.00506 (PMC6428702; doi:10.3389/fmicb.2019.00506)
Supplement: Supplementary file 6 [file Presentation_1.PPTX]

## Slide 1
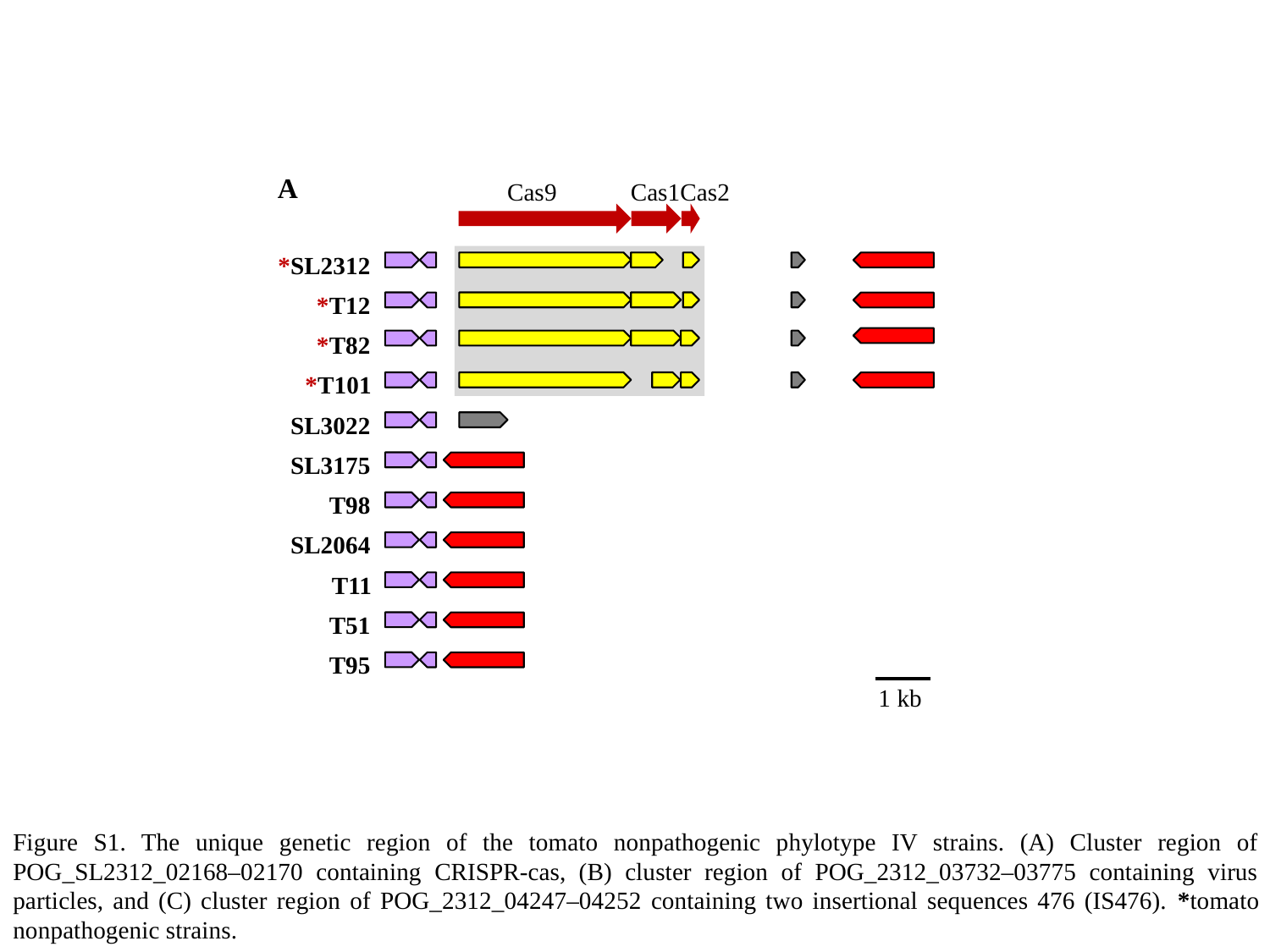

A
Cas9 Cas1Cas2
*SL2312
*T12
*T82
*T101
SL3022
SL3175
T98
SL2064
T11
T51
T95
1 kb
Figure S1. The unique genetic region of the tomato nonpathogenic phylotype IV strains. (A) Cluster region of POG_SL2312_02168–02170 containing CRISPR-cas, (B) cluster region of POG_2312_03732–03775 containing virus particles, and (C) cluster region of POG_2312_04247–04252 containing two insertional sequences 476 (IS476). *tomato nonpathogenic strains.

## Slide 2
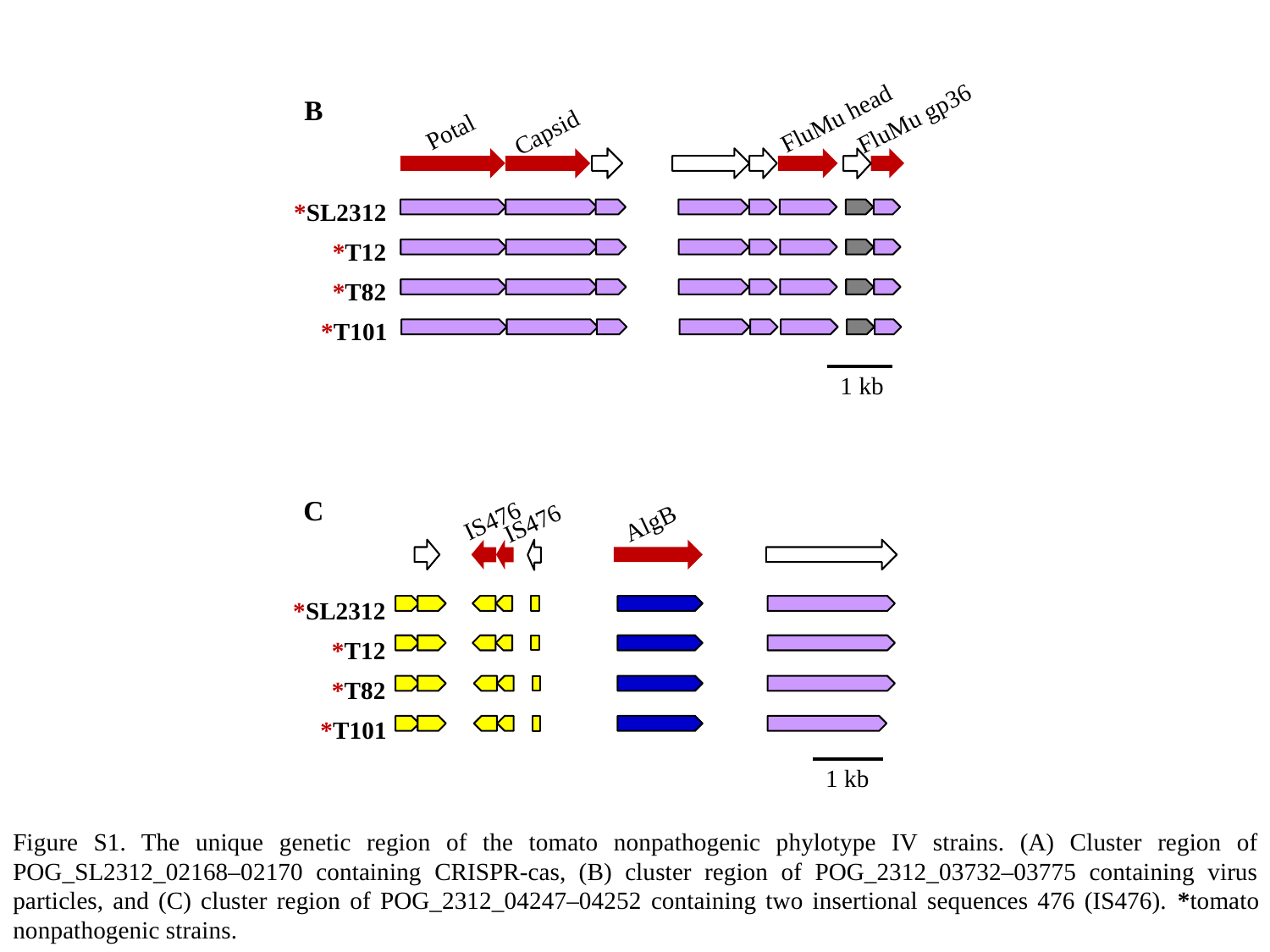

B
FluMu head
FluMu gp36
Capsid
Potal
*SL2312
*T12
*T82
*T101
1 kb
C
IS476
IS476
AlgB
*SL2312
*T12
*T82
*T101
1 kb
Figure S1. The unique genetic region of the tomato nonpathogenic phylotype IV strains. (A) Cluster region of POG_SL2312_02168–02170 containing CRISPR-cas, (B) cluster region of POG_2312_03732–03775 containing virus particles, and (C) cluster region of POG_2312_04247–04252 containing two insertional sequences 476 (IS476). *tomato nonpathogenic strains.

## Slide 3
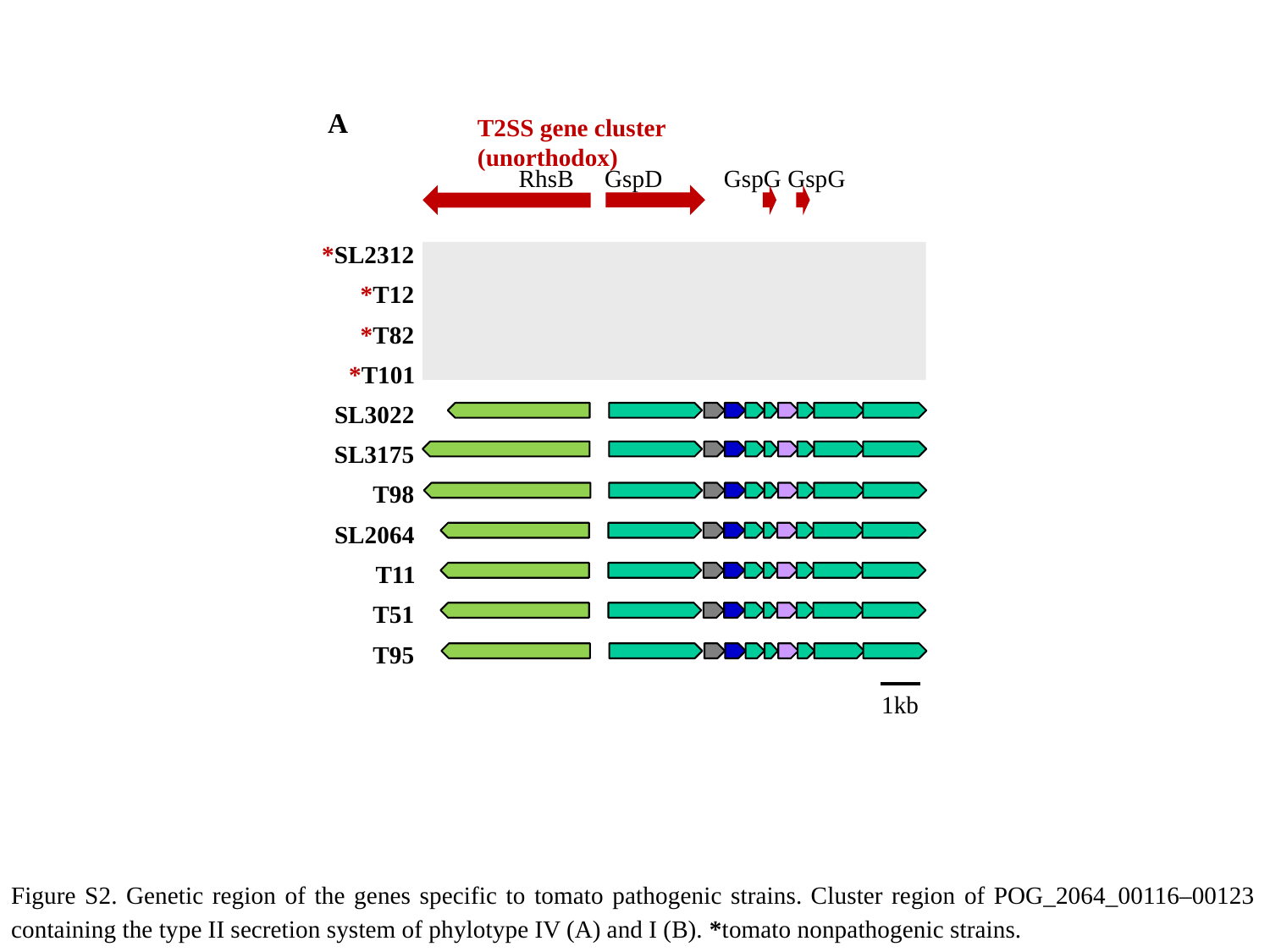

A
T2SS gene cluster (unorthodox)
RhsB GspD GspG GspG
*SL2312
*T12
*T82
*T101
SL3022
SL3175
T98
SL2064
T11
T51
T95
1kb
Figure S2. Genetic region of the genes specific to tomato pathogenic strains. Cluster region of POG_2064_00116–00123 containing the type II secretion system of phylotype IV (A) and I (B). *tomato nonpathogenic strains.

## Slide 4
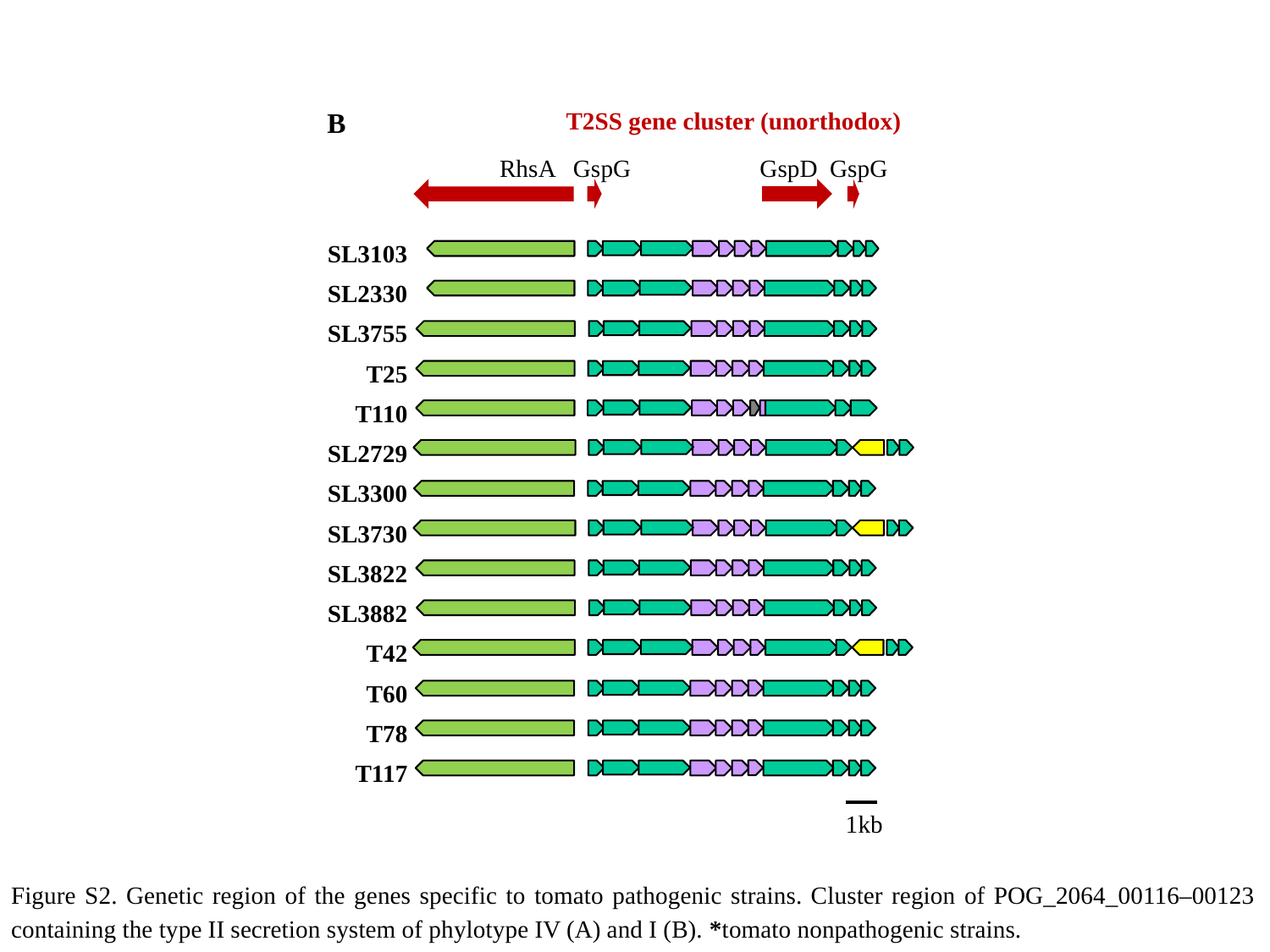

B
T2SS gene cluster (unorthodox)
RhsA GspG GspD GspG
SL3103
SL2330
SL3755
T25
T110
SL2729
SL3300
SL3730
SL3822
SL3882
T42
T60
T78
T117
1kb
Figure S2. Genetic region of the genes specific to tomato pathogenic strains. Cluster region of POG_2064_00116–00123 containing the type II secretion system of phylotype IV (A) and I (B). *tomato nonpathogenic strains.

## Slide 5
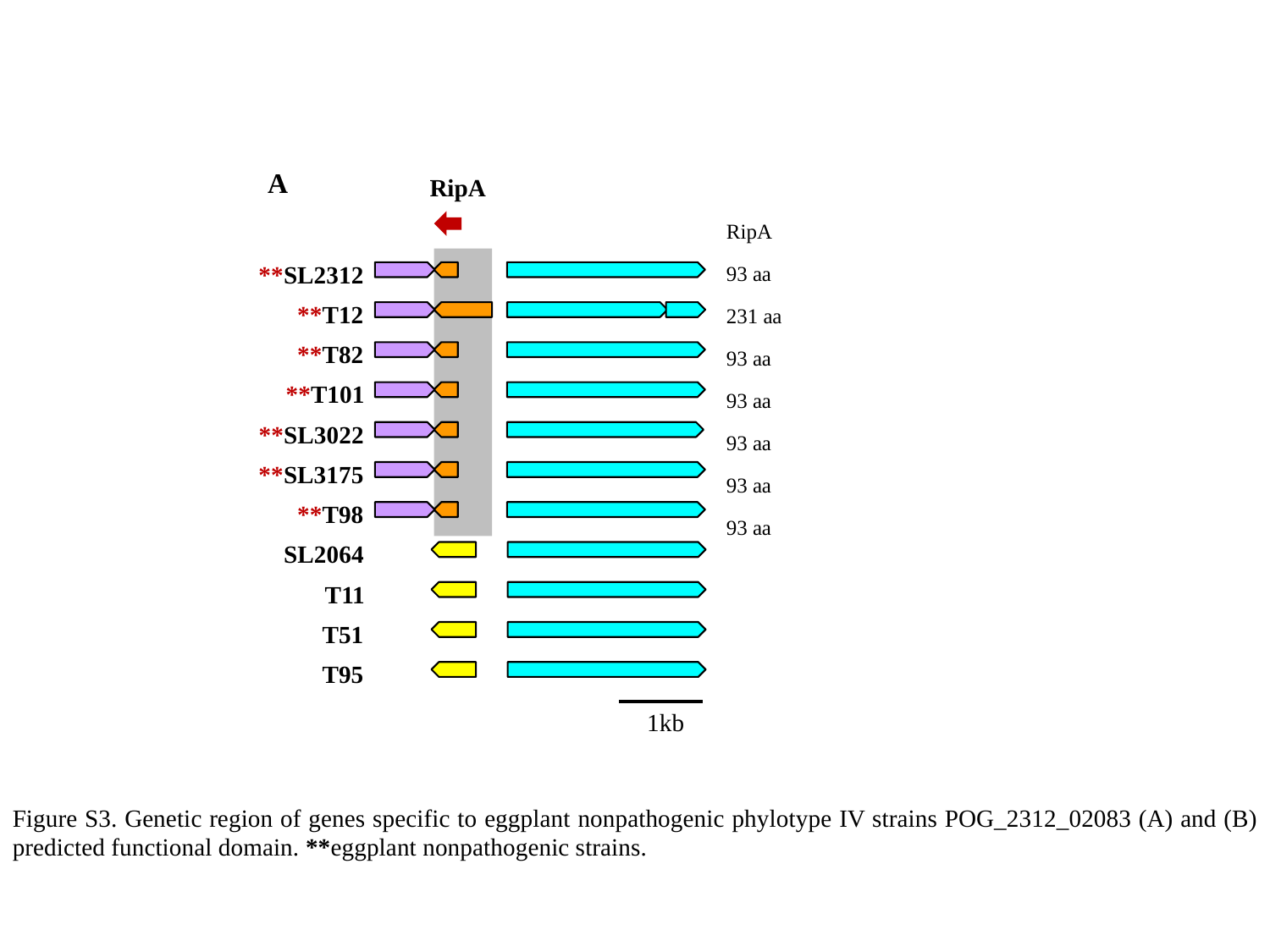

A
RipA
**SL2312
**T12
**T82
**T101
**SL3022
**SL3175
**T98
SL2064
T11
T51
T95
RipA
93 aa
231 aa
93 aa
93 aa
93 aa
93 aa
93 aa
1kb
Figure S3. Genetic region of genes specific to eggplant nonpathogenic phylotype IV strains POG_2312_02083 (A) and (B) predicted functional domain. **eggplant nonpathogenic strains.

## Slide 6
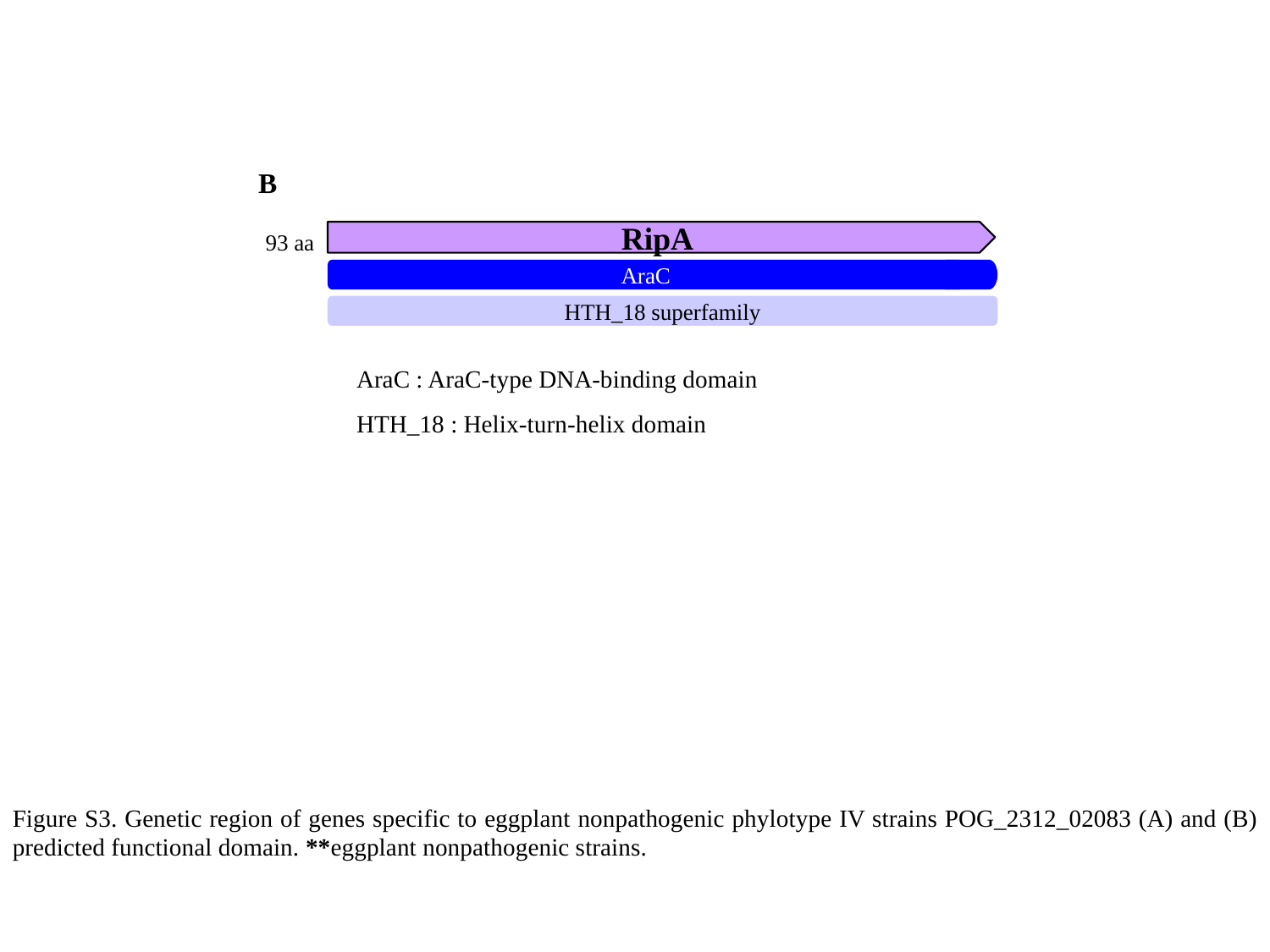

B
93 aa
RipA
AraC
HTH_18 superfamily
AraC : AraC-type DNA-binding domain
HTH_18 : Helix-turn-helix domain
Figure S3. Genetic region of genes specific to eggplant nonpathogenic phylotype IV strains POG_2312_02083 (A) and (B) predicted functional domain. **eggplant nonpathogenic strains.

## Slide 7
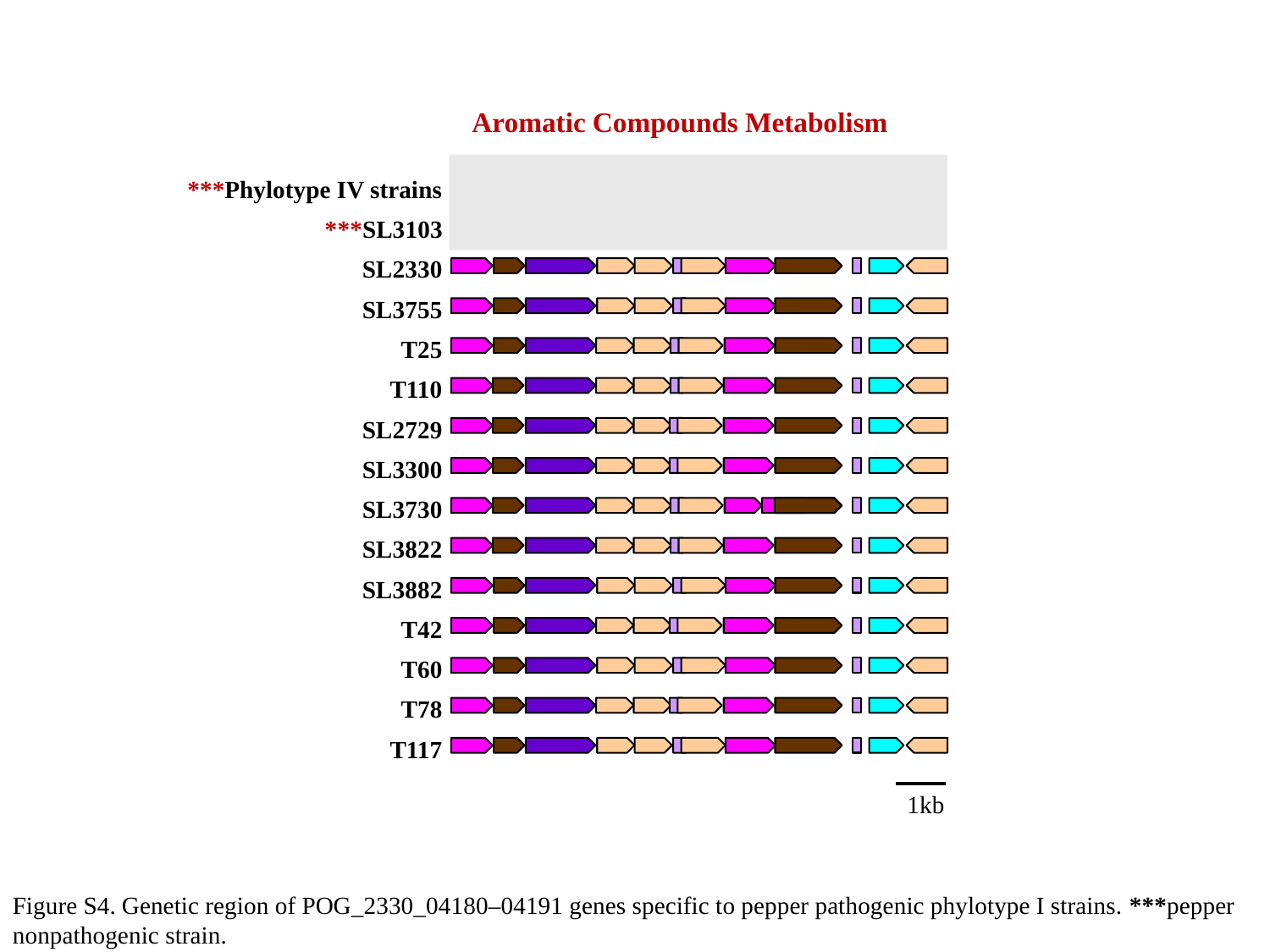

Aromatic Compounds Metabolism
***SL3103
SL2330
SL3755
T25
T110
SL2729
SL3300
SL3730
SL3822
SL3882
T42
T60
T78
T117
1kb
***Phylotype IV strains
Figure S4. Genetic region of POG_2330_04180–04191 genes specific to pepper pathogenic phylotype I strains. ***pepper nonpathogenic strain.

## Slide 8
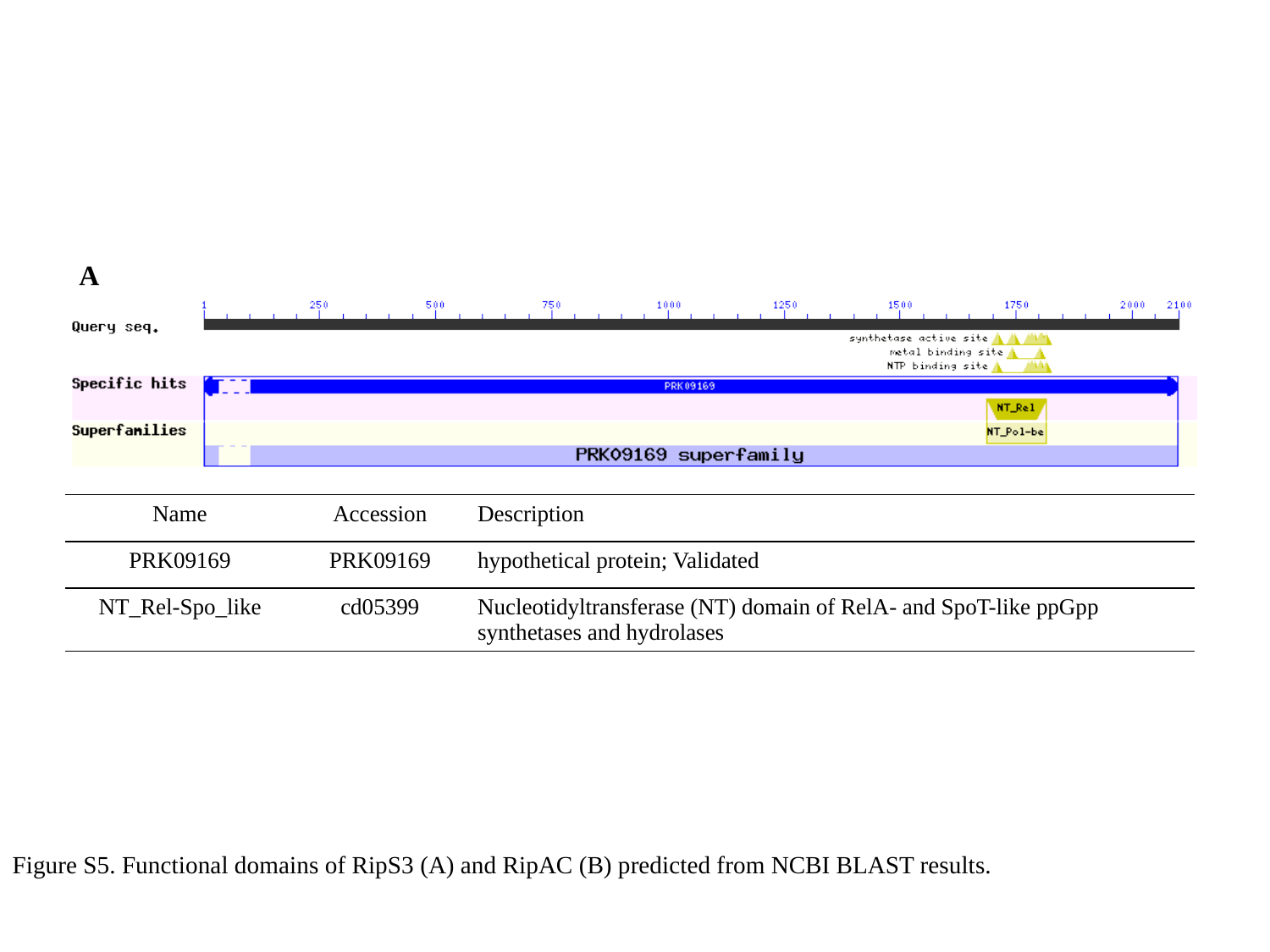

A
| Name | Accession | Description |
| --- | --- | --- |
| PRK09169 | PRK09169 | hypothetical protein; Validated |
| NT\_Rel-Spo\_like | cd05399 | Nucleotidyltransferase (NT) domain of RelA- and SpoT-like ppGpp synthetases and hydrolases |
Figure S5. Functional domains of RipS3 (A) and RipAC (B) predicted from NCBI BLAST results.

## Slide 9
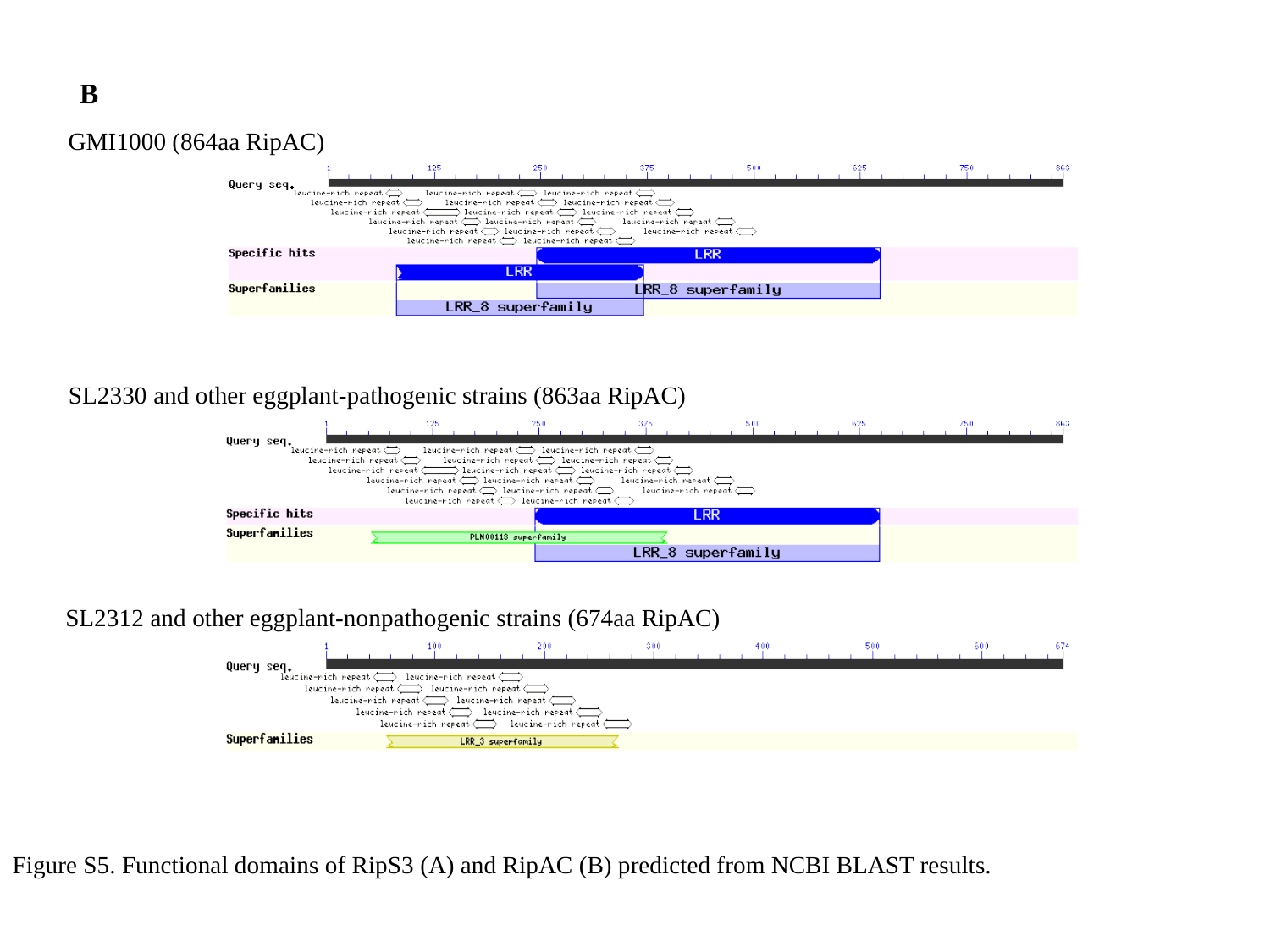

B
GMI1000 (864aa RipAC)
SL2330 and other eggplant-pathogenic strains (863aa RipAC)
SL2312 and other eggplant-nonpathogenic strains (674aa RipAC)
Figure S5. Functional domains of RipS3 (A) and RipAC (B) predicted from NCBI BLAST results.
